# Supplementary material for: Repurposing N-Doped Grape Marc for the Fabrication of Supercapacitors with Theoretical and Machine Learning Models
Source: Nanomaterials (Basel). 2022 May 27;12(11):1847. doi: 10.3390/nano12111847 (PMC9182344; doi:10.3390/nano12111847)
Supplement: Supplementary file 1 [file nanomaterials-12-01847-s001.zip › nanomaterials-1732789-supplementary.pdf]

nanomaterials

Supplementary Materials

Repurposing N-Doped Grape Marc for the Fabrication of Supercapacitors with Theoretical and Machine Learning Models

Kethaki Wickramaarachchi [1], Manickam Minakshi [1,*], S. Assa Aravindh [2], Rukshima Dabare [1], Xiangpeng Gao [1], Zhong-Tao Jiang [1] and Kok Wai Wong [1]

[1] College of Science, Health, Engineering & Education, Murdoch University, WA 6150, Australia; kethaki.wickramaarachchi@murdoch.edu.au (K.W.); rukshima_mech@yahoo.com (R.D.); x.gao@murdoch.edu.au (X.G.); z.jiang@murdoch.edu.au (Z.-T.J.); k.wong@murdoch.edu.au (K.W.)
[2] Nano and Molecular Systems Research Unit, University of Oulu, Pentti Kaiteran Katu 1, 90570 Oulu, Finland; assa.sasikaladevi@oulu.fi
* Correspondence: minakshi@murdoch.edu.au; Tel.: +61-89-3602017

Equations used for specific capacitance calculations:

$$C_{s,CD} = \frac{i\,\Delta t}{m\,\Delta v} \quad (1)$$

$$E = \frac{1}{2} C_{s,CD} \frac{\Delta v^2}{3.6} \quad (2)$$

$$\mathrm{P} = \frac{\mathrm{E}}{\Delta \mathrm{t}} \times 3600 \quad (3)$$

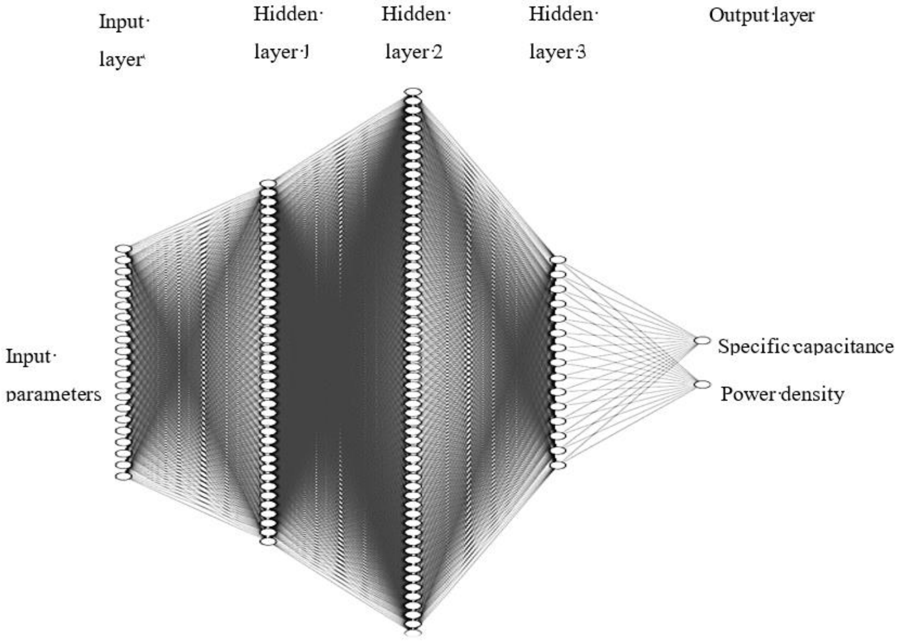

Figure S1. MLP model architecture for correlating the inputs with specific capacitance and the power density.

Equations to determine the ML model accuracy:

$$R^2 = 1 - \frac{\sum_{i=1}^{n}(y_i - u_i)^2}{\sum_{i=1}^{n}(y_i - \bar{u})^2} \quad (4)$$

$$MSE = \frac{\sum_{i=1}^{n}(y_i - u_i)^2}{n} \quad (5)$$

$$MAE = \sqrt{\frac{\sum_{i=1}^{n}|y_i - u_i|}{n}} \quad (6)$$

where n, y_i, u_i, $\bar{u}$ are, the number of data points, the value calculated from ML, the experimental value, and the average of all experimental data, respectively. To identify the best ML model, it should show the highest R^2, which would reach 1 for a perfect fit. At the same time, the MSE and an MAE should be the lowest. The smaller the MSE and MAE are, the closer the predicted and actual values.

To characterize the porous carbon from different biomass precursors, we postulate it is important to consider surface area, micropore volume, average pore diameter, and surface morphology properties. The more inputs to identify and differentiate the properties of the materials make the predictions more robust.[1] Further, the model results were compared with and without these morphology features. The effect of the changed current rate has been considered in this work as an electrochemical feature. The current rate directly affects the integral capacitance measured from the GCD curves, which generally decreases with an increasing rate. As another determining factor of the capacitance, the electrolyte properties, the hydrated radius of the ions, ion conductivity, and mobility have also been included as inputs. Thus 21 input parameters were considered as inputs for the MLP model. MLP model was built using the Scikit-learn package in Python. The relu' and 'linear' activation functions are used for the model, and the optimizer used is 'adam'. The MLP was trained for a maximum iteration of 2500 for this model. Out of the experimental data, 80% was used for training, and 20% was used to test the MLP model. The 5 - fold cross-validation splits the data into 5 equal-sized blocks and one block for testing and the other blocks for training for the model.[2] The results presented in this section consider the average of those 5 - fold cross-validation.

The reaction mechanism of the biochar in the presence of the KOH activating agent.[3]

$$6KOH + 2C \rightarrow 2K + 3H_2 + 2K_2CO_3 \quad (7)$$

$$K_2CO_3 + C \rightarrow K_2O + 2CO \quad (8)$$

$$K_2CO_3 \rightarrow K_2O + CO_2 \quad (9)$$

$$2K + CO_2 \rightarrow K_2O + CO \quad (10)$$

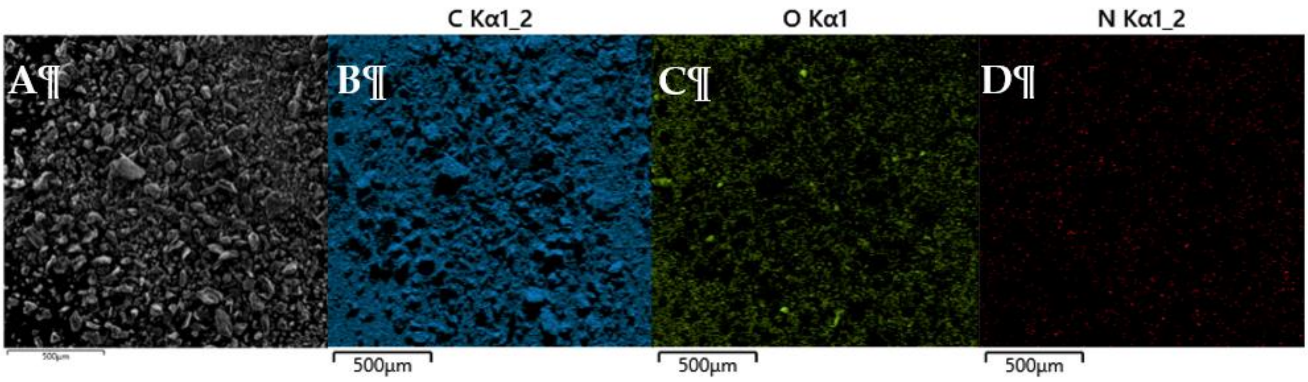

Figure S2. SEM coupled with EDS of KAC_{urea} (**A**) electron image, (**B–D**) elemental mapping of 'C', 'O' and 'N' respectively.

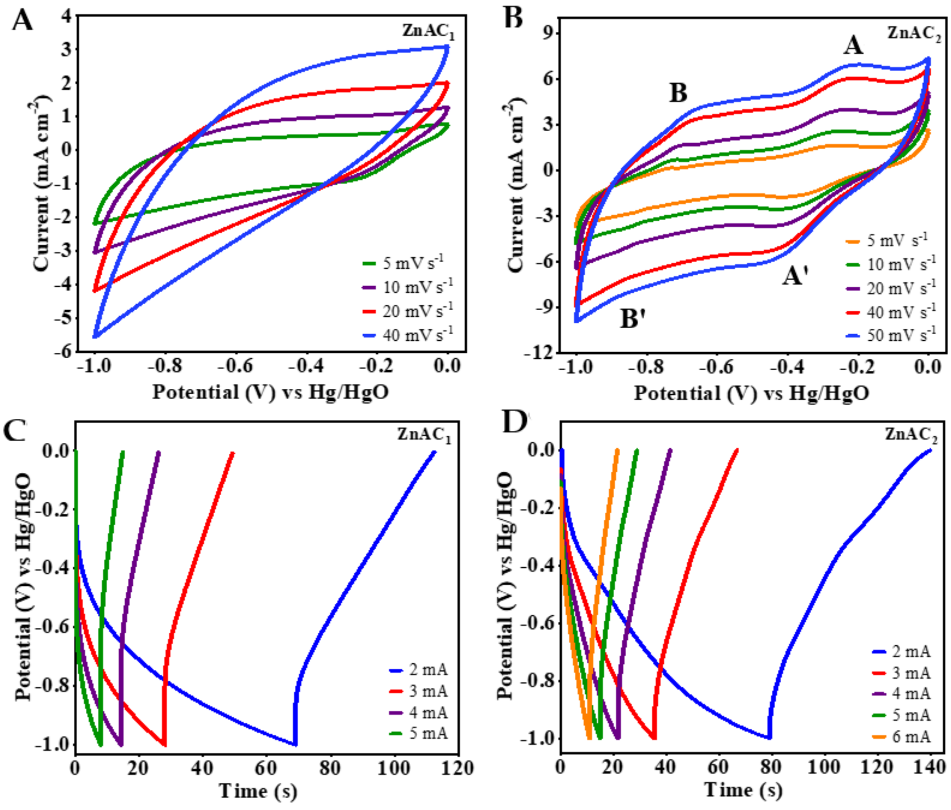

Figure S3. (**A**,**B**) Cyclic Voltammetry (CV), and (**C**,**D**) Galvanostatic charge-discharge curves of winery residue (WR)-derived AC: $ZnAC_1$ and $ZnAC_2$.

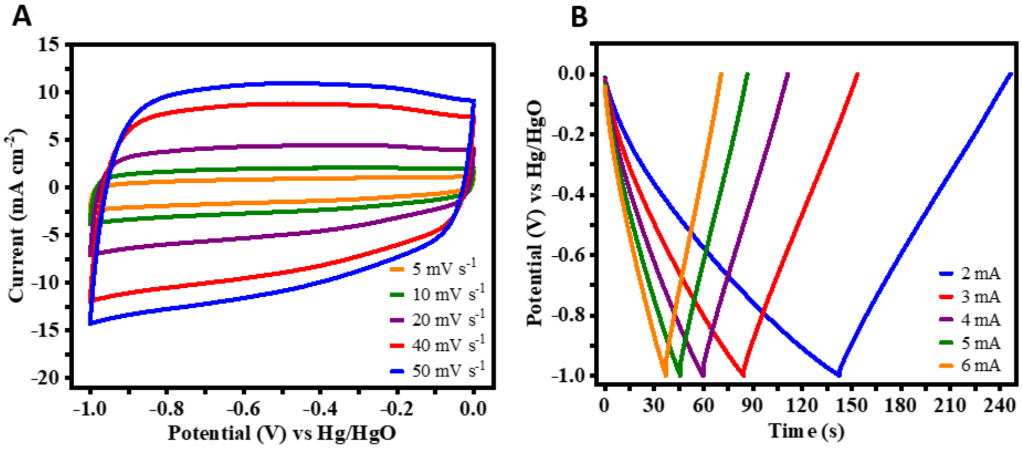

Figure S4. (**A**) Cyclic Voltammetry (CV) and (**B**) Galvanostatic charge-discharge curves of commercial AC to benchmark the values obtained for WR-derived carbon.

Comparison between the electrolytes:

The current response observed in the three electrolytes followed the order of NaOH > $NaClO_4$ > LiOH. When the electrolyte (cation/anion) is varied, the differences in the capacitances are observed due to ion mobility, hydrated ion radius, conductivity, and their effect on charge/ion exchange and diffusion.[4] The highest current response is seen in the NaOH compared to the LiOH electrolyte. The Li^+ ions have a larger hydration sphere

radius as it strongly interacts with $H_2O^{\delta-}$. Considering the effect of the anions, the presence of OH^- seems comparatively better than ClO_4^- on the electrochemical performance of KA-C_{urea}. The higher ionic conductivity, ionic mobility, and the smaller hydration sphere of OH^- ion led to the efficient charge propagation, as evidenced by CV and GCD measurements. Figure S3-C shows the highest capacitance retention given by NaOH over increasing current rates. The Nyquist plots for the three-electrode system are studied to further analyze the ionic diffusion and transport kinetics. It can be seen in Figure S3-D that all the plots demonstrate the contributions to the ideal capacitance observed for the three electrolytes with a -90° slope at the low-frequency region. In the middle frequency region, the Warburg impedance with a ~45° gradient inclined line is more understandable for LiOH, implying its slow ion transport kinetics. In summary, both the CV and GCD measurements concluded that the electrochemical performance of WR-derived activated carbon was significantly enhanced by the addition of N-dopant tested in Na-ion electrolyte as a promising and low-cost alternative to existing electrolytes.

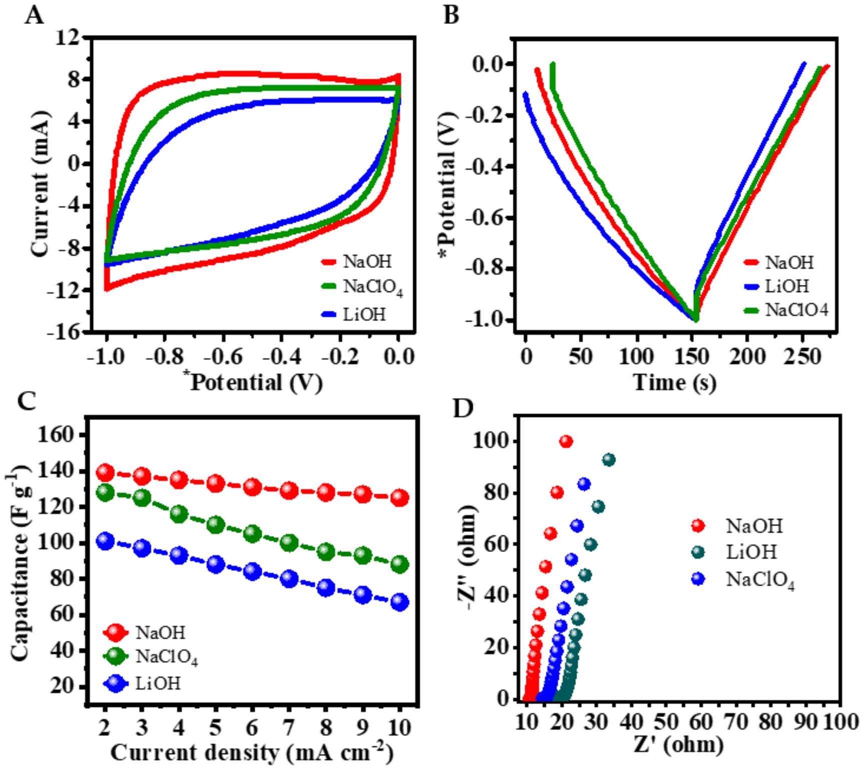

Figure S5. (**A**,**B**) Cyclic Voltammetry (CV) and (**C**,**D**) Galvanostatic charge-discharge curves of commercial AC to benchmark the values obtained for WR-derived AC.

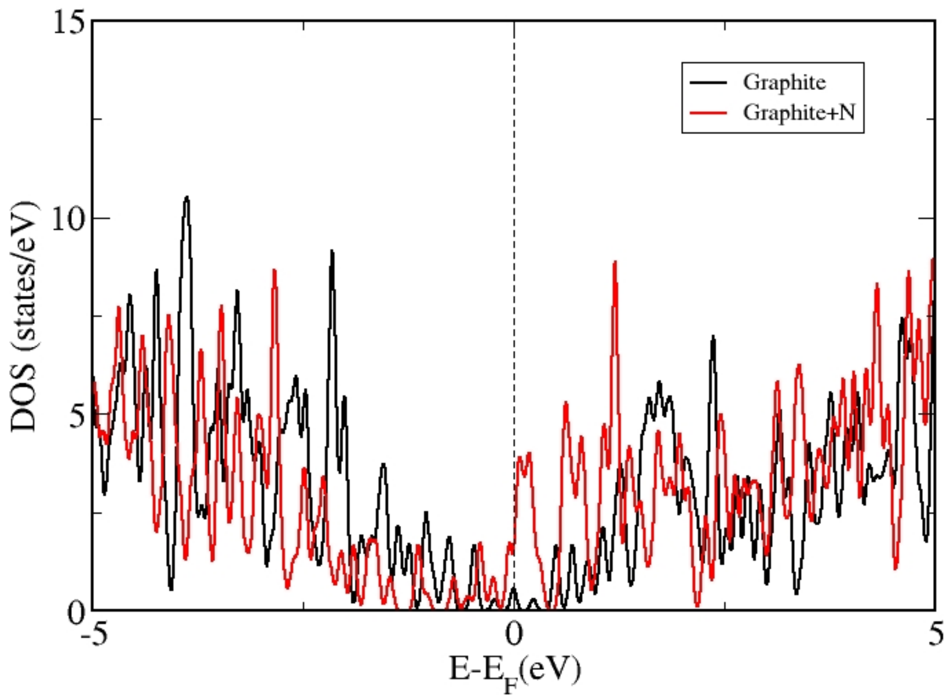

Figure S6. The total density of states (DOS) of the pristine and N doped graphite surfaces.

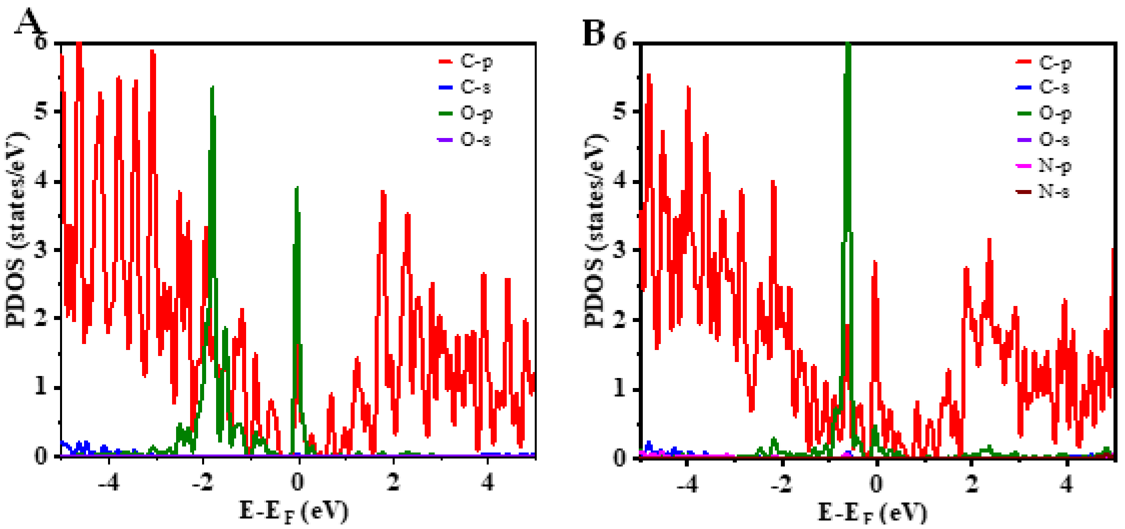

Figure S7. The PDOS calculated for (A) O adsorbed on pristine and (B) N-doped graphite surfaces.

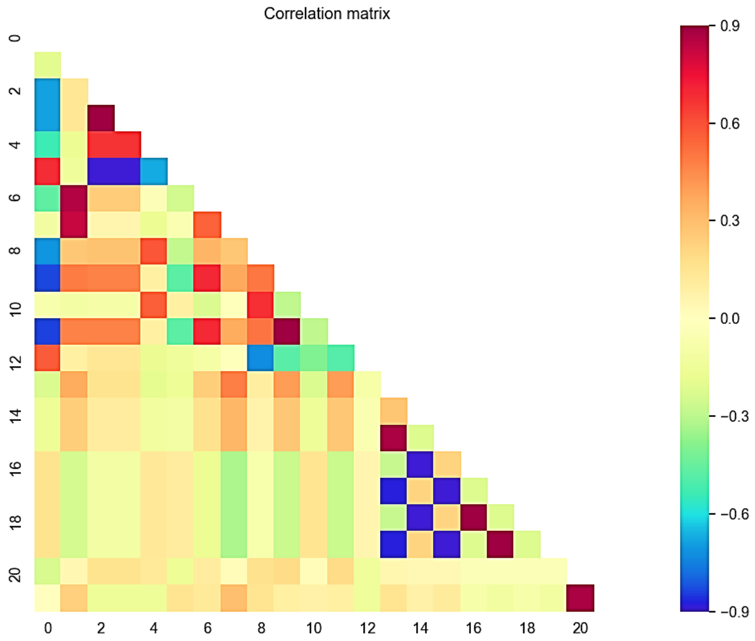

Figure S8. Pearsn Correlation Coefficient.

Table S1. Summary of inputs and outputs used for MLP model.

Inputs	Outputs
Activating agent type	
Dopant % in the precursor	
Carbonization temperature	
Carbonization time	
Activation temperature	
Activation time	
Porosity feature	
Surface average pore size	
BET surface area	
Micropore area	
External surface area	
Micropore volume	
BJH average pore diameter	Specific capacitance
Electrolyte type	
Cation hydrated ionic radius	Power density
Anion hydrated ionic radius	
Cation ionic conductivity	
Anion ionic conductivity	
Cation ionic mobility	
Anion ionic mobility	
Current rate	

References

1. Krishnamurthy, D.; Weiland, H.; Barati Farimani, A.; Antono, E.; Green, J.; Viswanathan, V. Machine Learning Based Approaches to Accelerate Energy Materials Discovery and Optimization. *ACS Energy Lett.* **2019**, *4* (1), 187–191.
2. Rogers, S.; Girolami, M. A First Course of Machine Learning, 2nd ed.; Chapman and Hall/CRC, **2016**.
3. Zhang, H.; Zhou, X. L.; Shao, L. M.; Lü, F.; He, P. J. Hierarchical Porous Carbon Spheres from Low-Density Polyethylene for High-Performance Supercapacitors. *ACS Sustain. Chem. Eng.* **2019**, *7* (4), 3801–3810.

4. Pal, B.; Yang, S.; Ramesh, S.; Thangadurai, V.; Jose, R. Electrolyte Selection for Supercapacitive Devices: A Critical Review. *Nanoscale Adv.* **2019**, *1* (10), 3807–3835.
